# Effect of Comedy Movies Watched During Transurethral Resection of the Prostate (TUR‐P) on Vital Signs and Postoperative Pain: A Randomized Controlled Trial

**DOI:** 10.1111/papr.70136

**Published:** 2026-03-07

**Authors:** Remziye Cici, Gülay Yilmazel, Mücahit Doğan, Muhammet Yaytokgil

**Affiliations:** ^1^ Faculty of Health Sciences Hitit University Çorum Turkey; ^2^ TC Saglık Bakanlıgı Mengucek Gazı Egitim Ve Arastırma Hastanesi Erzincan Turkey; ^3^ Bazekol Çiğli Hospital İzmir Turkey

**Keywords:** comedy movie, postoperative pain, prostate cancer, transurethral prostate resection, vital sign

## Abstract

**Aim:**

This study was conducted to determine how watching comedy movies affects vital signs and postoperative pain in patients during transurethral prostate resection.

**Methods:**

A randomized controlled trial model study was conducted at a hospital in Turkey. Ninety patients were included in the study (comedy: 30, documentary: 30, control: 30). Pain was assessed using the Visual Analog Pain Scale, while patient monitors recorded vital signs.

**Results:**

At the first measurement, the mean pain score in the comedy group was 2.0. Although this value was numerically the lowest among the groups, it was not statistically significant (*p* > 0.05). Across the three groups, baseline VAS values demonstrated similar ranges (95% CI: 2.73–3.41). In the 24th and 48th hour measurements, the pain was again at the lowest level in the comedy group (VAS 24 h 95% CI: 2.12–2.84; VAS 48 h 95% CI: 0.94–1.52; *p* < 0.001). Although a decrease was observed in the pulse rate of vital signs in all three groups in the postoperative measurements, it was determined that the lowest value was in the comedy group (pulse at operation start 95% CI: 79.92–84.47, 15th minute 95% CI: 73.79–87.69, 30th minute 95% CI: 46.03–68.70, end of surgery 95% CI: 71.75–76.14; *p* < 0.001). While a decrease in systolic blood pressure was observed in all three groups during and after surgery compared to the preoperative period, it was determined that the most significant decrease was in the comedy group (15th minute 95% CI: 117.12–124.82, 30th minute 95% CI: 74.46–94.95, end of surgery 95% CI: 116.54–123.46; *p* < 0.05).

**Conclusions:**

The study's results determined that comedy films significantly affected pain management and perioperative vital signs such as pulse and systolic blood pressure.

## 1 Introduction

Prostate diseases (benign prostatic hyperplasia/BPH, prostate cancer, and prostatitis) are a common health problem in men over the age of 40 [1, 2]. Transurethral Resection of the Prostate (TUR‐P) is used to treat these diseases. This method is applied to provide treatment to patients with BPH with a less invasive procedure if the prostate tissue is between 30 and 80 g. It is also preferred to relieve symptomatic urinary obstruction in advanced prostate cancer patients [3].

Spinal anesthesia is often preferred in TUR‐P procedures due to the effective sensory and motor block it creates [4]. However, since patients who undergo surgery with spinal anesthesia are conscious, they can hear the staff's conversations and other sounds, and the sounds heard can cause fear and anxiety in patients [5, 6]. This anxiety and fear are caused by an increase in sympathetic and endocrine stimuli, leading to an increase in respiratory rate and depth, heart rate, and blood pressure, and increased pain after surgery [7, 8].

Humor and laughter reduce feelings of tension, fear, anger, and anxiety, increase pain tolerance, regulate breathing, ensure better cardiac muscle functioning, increase circulatory rate, and reduce muscle tension [9, 10]. The literature mentions that watching a comedy movie before surgery has positive effects on the postoperative period [11], and therapeutic humor applied during painful procedures has positive effects on pain and anxiety during the procedure [12]. However, no application has been encountered during surgery.

Therefore, it is thought that comedy movies applied during the procedure to patients who underwent the TUR‐P procedure with spinal anesthesia may affect vital signs and postoperative pain. Therefore, the study was conducted to determine the effect of comedy movies' vital signs and postoperative pain in patients during transurethral prostate resection.

## 2 Methods

The research is a pre‐test and post‐test randomized controlled real‐trial model study conducted at a hospital in northern Turkey between June 2022 and March 2024. This study followed the steps of the Consolidated Standards of Reporting Trials (CONSORT) statement.

### 2.1 Participants

**FIGURE 1:**
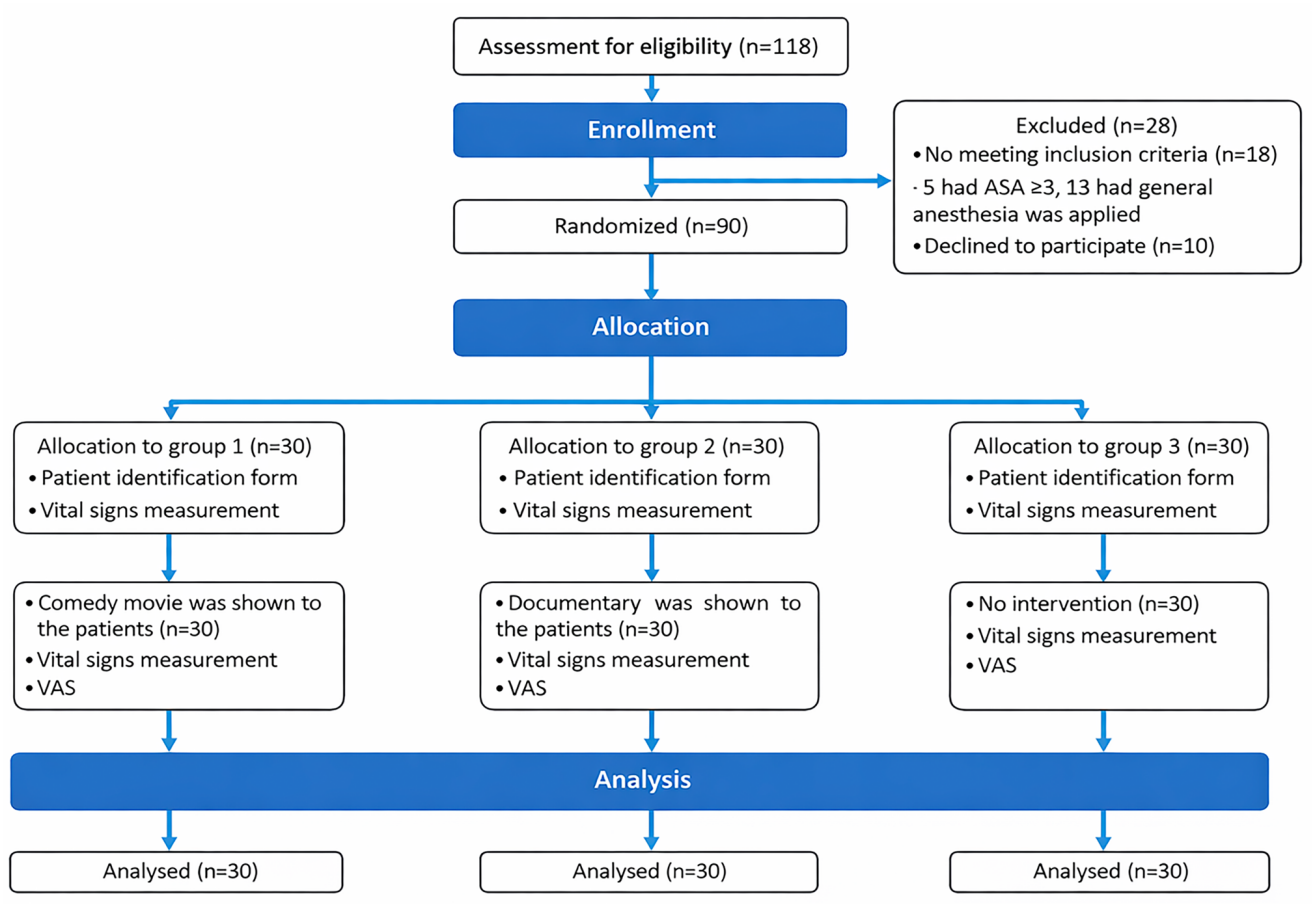
Sample groups and patient selection diagram.

The research was conducted with three groups: the comedy group, which watched funny videos during TUR‐P surgery in a randomized controlled manner; the documentary group, which watched documentaries to reveal the determining factors other than comedy; and those who did not watch any videos in the control group. The study's sample size was calculated using the G*Power 3.1.9.7 program. The sample calculation was made for One‐Way ANOVA analysis, considering the three‐group (experimental and control group) research design. In the calculation, the effect size was calculated as 0.40 (*f* = 0.40), a 5% margin of error (α = 0.05), and 90% power (1‐β = 0.90), and the number of samples for each group was calculated as 28 [13, 14]. According to the power analysis results, the control and experimental groups should have included *n* = 84 patients. Considering the possibility of data loss, the number of samples for each group was increased by approximately 10%. The study's reach was *n* = 118 patients. After the exclusion of *n* = 18 patients who did not meet the inclusion criteria (five patients had ASA > 3, 13 patients had general anesthesia applied), and *n* = 10 patients who did not agree to participate in the study, the sample of the study consisted of *n* = 90 patients, including *n* = 30 patients in each group (Figure 1). Patients were assigned to groups by lottery method.

The inclusion and exclusion criteria for this study are listed below. The inclusion included volunteer patients aged 18 and over who were scheduled for TUR‐P, had no vision, speech, hearing, or mental problems, were ASA (American Society of Anesthesiologists) classified as I, II, or III, and underwent spinal anesthesia. Patients who underwent general anesthesia and were ASA classified as over III were excluded from the study.

### 2.2 Data Collection and Data Collection Tools

Data were collected using a questionnaire prepared by the researchers. The questionnaire was prepared in three parts (Patient Identification Form, Vital Signs Record Form, and Visual Analog Pain Scale).

### 2.3 Patient Identification Form

This form includes questions about age, education, marital status, presence of chronic disease, previous surgery and type of anesthesia, the pain level in the previous surgery (Visual Analog Pain Scale), and ASA score. Prostate Specific Antigen (PSA) value, prostate volume, biopsy history and result, preop/postop hemoglobin values, duration of the operation, complications, transfusion status, and urinary catheter insertion time were also included in this section.

### 2.4 Vital Signs Record Form

The second part included the pulse, respiration, and blood pressure values obtained by measurement of the patients.

### 2.5 Visual Analog Pain Scale (VAS)

This scale allows patients to mark their pain on a ruler, with painlessness written on the left and the most severe pain (unbearable pain) written on the right. High scores indicate high pain intensity. 1–4 points are interpreted as mild pain, 5–6 points as moderate pain, and 7–10 points as severe pain [15].

The Patient identification form was applied to all patients in the clinic before going to the operating room. All patients underwent spinal anesthesia only, and no sedation was administered at any stage of the procedure. The spinal anesthesia procedure was standardized. A dose of 15 mg hyperbaric bupivacaine was administered to all patients by the same anesthesia team. The values of the vital signs (pulse, respiratory rate, systolic and diastolic blood pressure) included in the study data were recorded for all three groups when they were placed on the table before the operation, at the beginning of the procedure, at the 15th and 30th minutes of the operation, and at the end of the operation. After the operation, the patients were evaluated for pain intensity using the visual analog scale. Pain measurements were made three times for all three groups. The first measurement was made on the first day after the anesthesia wore off, the second measurement was made at the 24th hour of the operation, and the third and final measurement was made at the 48th hour.

### 2.6 Comedy Group

This group was shown a video of Charlie Chaplin movie episodes, prepared silently so that the sound would not distract during the operation. The video was started after the first vital signs were measured on the operating table just before the procedure and was watched by the patients without interruption throughout the operation. The video was shown on a screen mounted on the ceiling so patients could see it without disrupting the staff's workflow.

### 2.7 Documentary Group

The study included a group that was shown a documentary to determine the effectiveness of distraction. Before the surgery began, the patients were shown a silent, nonviolent documentary throughout the surgery. Before the surgery began, the patients were shown a silent, nonviolent documentary throughout the procedure, consisting of a compilation of nonverbal nature‐themed clips such as birds, forests, and landscapes merged into a single video.

The video was started after the first vital signs were measured on the operating table just before the procedure and was watched by the patients without interruption throughout the operation. The video was shown on a screen mounted on the ceiling so patients could see it and not interfere with the staff's workflow.

### 2.8 Control Group

No additional interventions were made during the surgery other than the procedures performed in the hospital. Vital signs and pain measurements were taken simultaneously as the other two groups.

To prevent individual preference bias, neither group was given an option to select the video and patients were not asked whether they liked the videos. Both the comedy and the documentary videos were compiled into one standardized silent video for all patients and were shown throughout the surgery without interruption.

### 2.9 Statistical Analysis

The data of the study were evaluated with the SPSS 22.0 program. In the evaluation of the data, the conformity of the variables to normal distribution was examined with the Kolmogrov‐Smirnov test, and it was determined that they did not show normal distribution. Percentage, mean, standard deviation, and median were used to analyze descriptive variables. Pearson Chi‐square test, Kruskal Wallis test, and One‐Way ANOVA test were performed to analyze categorical variables. In the analyses, the *p* < 0.05 value was considered statistically significant.

## 3 Results

### 3.1 Baseline Demographics and Clinical Characteristics

The mean age of the patients participating in the study was 69.3 ± 8.9 years in the comedy group, 65.9 ± 9.9 years in the documentary group, and 69.8 ± 7.3 years in the control group, and there was no significant difference between the groups (*p* > 0.05). 70% of all patients participating in the study were primary school graduates. The participants' chronic disease rate was 60%, 35.2% in the comedy group, 31.5% in the documentary group, and 33.3% in the control group (*p* > 0.05). Body mass index (BMI) did not show a statistically significant difference between the groups (*p* > 0.05) (Table 1).

The mean VAS score in the previous surgeries did not significantly differ between the groups (*p* > 0.05, 95% CI: 2.73–3.41) (Table 1).

The median ASA score was 2.5 in all three groups, with no statistically significant difference (*p* > 0.05). While 51.1% of the patients had previously experienced surgery under local/general anesthesia, the rate of those with anesthesia experience was 66.7% in the comedy group and 56.7% in the documentary group (*p* < 0.05). There was no significant difference between the groups regarding biopsy history and biopsy results (*p* > 0.05) (Table 1).

### 3.2 Data on Surgical Treatment and Pain Levels

Table 2 shows the distribution of information regarding the surgical treatment applied to the patients. No significant difference was found between the groups in terms of prostate PSA (*p* > 0.05, 95% CI: 3.12–5.57) and volume (*p* > 0.05, 95% CI: 50.34–58.54).

No significant difference was found between the groups in operation time (*p* > 0.05, 95% CI: 41.78–47.79) or catheter removal time (*p* > 0.05, 95% CI: 3.14–3.32).

While the scores obtained from the VAS did not differ between the groups on the first day (*p* > 0.05, 95% CI: 2.73–3.41), the VAS scores at the 24th (*p* < 0.05, 95% CI: 2.12–2.84) and 48th hours (*p* < 0.05, 95% CI: 0.94–1.52) were significantly higher in the documentary group. The number of patients who developed complications related to the operation was 3.3%, and all patients who developed complications were in the documentary group (*p* < 0.05).

### 3.3 Data on Vital Signs

Table 3 compares vital signs between the groups. When the preoperative vital signs of the study patients were compared between the groups, no statistically significant difference was observed in pulse rate, systolic and diastolic blood pressure values (p > 0.05), while a significant difference was determined between respiratory rates. The median respiratory rate was significantly higher in the documentary group before surgery (*p* < 0.05, 95% CI: 14.45–15.43). The median respiratory rate in the documentary group was 16.5 immediately before the procedure, significantly higher than in the comedy group (*p* < 0.05, 95% CI: 14.96–16.22).

The median pulse rate in the documentary and control groups was 78.6/81.1 beats at the 15th minute (*p* < 0.05, 95% CI: 73.79–87.69) and 75.7/76.5 beats at the end of the surgery (*p* < 0.05, 95% CI: 71.75–76.14), significantly higher than in the comedy group (*p* < 0.05).

At the 15th minute of surgery, the median systolic blood pressure was 123.5 mmHg in the control group, 122.3 mmHg in the documentary group, and 116.0 mmHg in the comedy group (*p* < 0.05, 95% CI: 117.12–124.82). At the end of the surgery, the median systolic blood pressure was 116.9 mmHg in the comedy group, 127.5 mmHg in the control group, and 117.2 mmHg in the documentary group (*p* < 0.05, 95% CI: 116.54–123.46). There was no significant difference between the groups regarding diastolic blood pressure (*p* > 0.05, Pre‐op 95% CI: 77.82–80.98, End of surgery 95% CI: 73.37–93.71).

## 4 Discussion

In this study, there was no difference between the groups regarding demographic data and laboratory values such as prostate PSA, prostate volume, and preoperative hemoglobin, indicating that the three groups were similar before the intervention (Tables 1 and 2).

**TABLE 1:** Baseline patient demographics and clinical characteristics.

Variables		Group comedy (*n* = 30)		Group documentary (*n* = 30)		Group control (*n* = 30)		Statistical analysis		
		*ꭓ* ± SD		*ꭓ* ± SD		*ꭓ* ± SD				
Age		69.3 ± 8.9		65.9 ± 9.9		69.8 ± 7.3		F: 1.693/0.190		
VAS score in previous surgery (median)		1.0		1.0		0.0		KW: 4.014/0.134		
ASA score (median)		2.5		2.5		2.5		KW: 0.818/0.664		
BMİ (body mass index)		27.8 ± 3.6		26.2 ± 3.2		26.8 ± 1.7		F: 0.920/0.616		
								**Total**		**Statistical analysis (*χ* ^2^)**
		***n***	**%**	***n***	**%**	***n***	**%**	***n***	**%**	
Education level	Primary education	21	33.3	20	31.7	22	34.9	63	70.0	0.317/0.853
	High school and above	9	33.3	10	37.1	8	29.6	27	30.0	
Chronic disease	Yes	19	35.2	17	31.5	18	33.3	54	60.0	0.278/0.870
	No	11	30.6	13	36.1	12	33.3	36	40.0	
Operation experience	Yes	19	41.3	17	37.0	10	21.7	46	51.1	5.958/0.051
	No	11	25.0	13	29.5	20	45.5	44	48.9	
Previous type of anesthesia	Local	9	45.0	8	40.0	3	15.0	20	22.2	6.684/0.154
	General	10	38.5	9	34.6	7	26.9	26	28.9	
	No anesthesia	11	25.0	13	29.5	20	45.5	44	48.9	
Biopsy history	Yes	5	20.8	11	45.8	8	33.3	24	26.7	3.068/0.216
	No	25	27.9	19	28.8	22	33.3	66	73.3	
Biopsy result	Not done	25	37.9	19	28.8	22	33.3	66	77.3	4.470/0.346
	Benign	5	21.7	10	45.5	8	34.8	23	25.6	
	Malign	0	0.0	1	100.0	0	0.0	1	1.1	

Abbreviations: F, one‐way anova test; KW, Kruskal Wallis test; SD, standard deviation; *χ* ^2^, Pearson Chi‐square test.

**TABLE 2:** Distribution of information regarding surgical treatment applied.

Variables		Group comedy (*n* = 30)		Group documentary (*n* = 30)		Group control (*n* = 30)		Statistical analysis		
		Median		Median		Median				
Prostate PSA		2.0		2.1		1.3		1.374/0.503^a^		
Prostate volume		45.0		50		50.0		2.097/0.351^a^		
Preoperative Hg		14.6		14.0		14.0		2.583/0.275^a^		
Postoperative Hg		13.8		13.2		13.7		4.540/0.103^a^		
Operation time (minute)		45.0		40.0		40.0		3.374/0.185^a^		
Probe emergence time (days)		3.0		3.0		3.0		1.037/0.595^a^		
VAS first day		2.0		3.0		3.0		2.698/0.260^a^		
VAS 24 h		1.0		4.0		2.0		24.417/0.000** ^a^		
VAS 48 h		0		2.0		1.0		20.246/0.000** ^a^		
								**Total**		**Statistical analysis**
		***n***	**%**	***n***	**%**	***n***	**%**	***n***	**%**	
Operation complication	Yes	0	0.0	3	100.0	0	0.0	3	3.3	6.207/0.045* ^b^
	No	30	34.5	27	31.0	30	34.5	87	96.7	

*Note:* *p < 0.05; **p < 0.001.

Abbreviations: Hg, hemoglobin; PSA, prostate specific antigen.

^a^ KW/Kruskal Wallis test.

^b^ *χ* ^2^/Pearson Chi‐square test.

The patient's pain levels were lower in the comedy group than in the other groups in all three measurements (Table 2). No study was found in the literature on the relationship between the use of comedy movies during surgery and pain. However, it was determined that the comedy movie watched by oncological surgery patients before surgery had a positive effect on the level of pain and anxiety after surgery [11]. Although it was not a surgical intervention, a study conducted with dialysis patients found that watching a comedy movie during the procedure reduced headaches [16]. In the study conducted by Kurudirek et al., it was determined that therapeutic humor applied using a clown during intrathecal chemotherapy reduced children's pain and anxiety [12]. Again, in many studies conducted with children, it has been reported that clown visits and comedy reduce pain and anxiety [17, 18, 19, 20]. Despite the population difference, this literature information supports our study. Unlike our study, Elmalı and Akpınar stated in their study that pain decreased during and after watching a video and then returned to its previous level [21]. There is information in the literature that laughing and watching funny videos strengthen the immune system, help muscles relax, reduce anxiety, and stimulate endorphin release [9, 10, 17]. Although the effect of anxiety on pain was not examined in our study, this information may be why the effect of comedy lasted longer, as in our study. In addition, the fact that the pain level was determined to be the highest in three measurements in the documentary group (Table 2) indicates that comedy is more effective in reducing the pain level without diverting attention.

The pain and high anxiety experienced are related to complications in the perioperative period [22, 23]. TUR‐P intervention has complications such as bleeding, prolonged catheterization, and coagulation problems [24]. In this study, similar to the literature, the complication rate during and after surgery was shallow in all patients. However, it is pleasing and exciting that no complications were detected in the comedy group (Table 2).

The positive effect of watching funny videos and humor on anxiety [7, 8] suggests that it may also affect vital signs, which are physiological symptoms of anxiety. Literature supports this and mentions the positive effects of anxiety on respiration, blood pressure, and pulse [9, 10]. A study conducted with children determined that training with a clown before surgery reduced the pulse rate after surgery [20]. In the patient group included in this study, the decrease in pulse rate just before surgery was only in the group that watched funny videos. The most significant decrease at the end of surgery was again in the comedy group, which supports the literature (Table 3). In the study of Genç and Sarıtaş on patients scheduled for oncological surgery, the positive effect of comedy movies on postoperative blood pressure was mentioned [25]. Another study conducted with dialysis patients reported that watching a comedy movie reduced the rate of hypertension during the procedure [16]. A study involving children and their parents determined that the group that received preoperative education with a clown nurse had lower postoperative systolic blood pressure than the group that received standard education [20]. In this study, it is thought that the decrease in systolic and diastolic blood pressure in all three groups at the end of surgery was due to the relief created by the end of the surgery. However, the fact that the decrease in systolic blood pressure was the highest in the comedy group among all three groups at the 15th and 30th minutes and in the postoperative measurements may indicate the effectiveness of comedy, supporting the literature.

**TABLE 3:** Comparison of vital signs between groups.

Vital signs	Group comedy (*n* = 30)	Group documentary (*n* = 30)	Group control (*n* = 30)	Statistical analysis
	Median	Median	Median	
*Pulse (beats/minute)*				
Pre‐operation	76.0	82.0	82.2	2.878/0.237^a^
The moment the operation starts	73.5	85.0	84.6	13.891/0.001* ^a^
15th minute	66.0	78.6	81.1	24.757/0.000** ^a^
30th minute	66.9	78.2	77.8	14.336/0.001* ^a^
End of surgery	66.0	75.7	76.5	16.186/0.000** ^a^
*Respiratory rate (minute)*				
Pre‐operation	13.9	15.6	14.3	12.586/0.002* ^a^
The moment the operation starts	14.5	16.5	14.9	7.269/0.026* ^a^
15th minute	13.7	15.4	14.1	9.590/0.080^a^
30th minute	14.2	15.6	14.5	2.693/0.026*^a^
End of surgery	14.3	15.5	14.3	6.305/0.043* ^a^
*Systolic blood pressure (mmHg)*				
Pre‐operation	137.5	130.6	137.3	3.950/0.139^a^
The moment the operation starts	132.2	129.5	135.5	4.060/0.131^a^
15th minute	116.0	122.3	123.5	12.041/0.002* ^a^
30th minute	117.8	118.1	128.1	6.646/0.036* ^a^
End of surgery	116.9	117.2	127.5	12.264/0.002* ^a^
*Diastolic blood pressure (mmHg)*				
Pre‐operation	78.6	78.9	79.6	0.207/0.902^a^
The moment the operation starts	77.8	79.5	79.4	0.719/0.698^a^
15th minute	72.3	75.6	77.4	0.398/0.819^a^
30th minute	72.6	73.5	75.5	1.983/0.371^a^
End of surgery	72.6	97.7	75.4	1.761/0.415^a^

*Note:* *p < 0.05; **p < 0.001.

^a^ KW/Kruskal Wallis test.

Our study's limitation is that the comedy movie was watched only by prostate patients, and anxiety that could affect vital signs and pain was not measured.

## 5 Conclusions

As a result, it was determined that the comedy movie reduced the level of postoperative pain and caused a significant decrease in the vital signs of pulse and systolic blood pressure during surgery, but it was ineffective on respiration. It was also determined that comedy was more effective in reducing pain and vital signs of life than documentary (diverting attention).

Watching funny videos can be a cost‐effective, accessible, and reliable method of regulating vital signs during surgery and reducing postoperative pain. In addition, it can be recommended that comedy movies be watched in different surgical intervention groups in future studies. However, it is recommended that future studies also assess anxiety levels to more comprehensively determine the relationship between humorous content exposure and pain.

## Author Contributions

Conception and Design: R.C., M.Y., and G.Y.; Acquiring the data: M.D.; Analysis: G.Y.; Literature review: R.C.; Reviewing the manuscript: R.C.; Final approval: R.C., G.Y., M.D., and M.Y.

## Ethics Statement

The study was conducted following the Declaration of Helsinki. Before starting the study, permissions were obtained from the Hitit University Clinical Research Ethics Committee (Decision No: 2022‐29) and the clinic where the study would be conducted on 15.03.2022. Individuals participating in the study were informed that they could withdraw from the study at any time, and their verbal and written consents were obtained.

## Conflicts of Interest

The authors declare no conflicts of interest.

## Data Availability

The data that support the findings of this study are available on request from the corresponding author. The data are not publicly available due to privacy or ethical restrictions.

## References

[1] X. Shu‐Jie , C. Di , and J. Qi , “An Overview of Prostate Diseases and Their Characteristics Specific to Asian Men,” Asian Journal of Andrology 14 (2012): 458–464, 10.1038/aja.2010.137. 22306914 PMC3720159

[2] R. Vidmar , G. Marcq , V. Flamand , et al., “Salvage Radical Prostatectomy for Recurrent Prostate Cancer. Morbidity, Oncological and Functional Results,” Progrès en Urologie 27 (2017): 458–466, 10.1016/j.purol.2017.05.005. 28576424

[3] J. Klein , A. S. Gözen , M. Fiedler , P. Rieker , and J. J. Rassweiler , “TUR‐P,” in Practical Tips in Urology, ed. A. Rané , B. Turna , R. Autorino , and J. Rassweiler (Springer, 2017), 479–485, 10.1007/978-1-4471-4348-2_49.

[4] A. Casati , E. Moizo , C. Marhetti , and F. Vinciguerra , “A Prospective, Randomised, Double‐Blind Comparison of Unilateral Spinal Anesthesia With Hyperbaric Bupivacaine, Ropivacaine, or Levobupivacain for Inguinal Herniorrhapyh,” Anesthesia and Analgesia 99 (2004): 1387–1392. 15502035 10.1213/01.ANE.0000132972.61498.F1

[5] P. Kukreja , K. Talbott , L. MacBeth , E. Ghanem , and A. Sturdivant , “Effects of Music Therapy During Total Knee Arthroplasty Under Spinal Anesthesia: A Prospective Randomized Controlled Study,” Cureus 12 (2020): 2–9, 10.7759/cureus.7396. PMC7179990 32337122

[6] H. Kaur , N. Saini , G. Singh , A. Singh , A. Dahuja , and R. Kaur , “Music as an Aid to Allay Anxiety in Patients Undergoing Orthopedic Surgeries Under Spinal Anesthesia,” Noise & Health 24 (2022): 7–12, 10.4103/nah.nah_58_21. 35645134 PMC9239141

[7] M. Iwasaki , M. Edmondson , A. Sakamoto , and D. Ma , “Anesthesia, Surgical Stress, and “Long‐Term” Outcomes,” Acta Anaesthesiologica Taiwanica 53 (2015): 99–104, 10.1016/j.aat.2015.07.002. 26235899

[8] A. Sidar , Ö. Dedeli , and A. İ. İşkesen , “The Relationship Between Anxiety, Pain Distress and Pain Severity Before and After Open Heart Surgery in Patients [Açık Kalp Cerrahisi Öncesi Ve Sonrası Hastaların Kaygı Ve Ağrı Distresi: Ağrı Düzeyi Ile Ilişkisinin Incelenmesi],” Turkish Journal of Medical and Surgical 4 (2013): 1–8.

[9] R. A. Martin , “Sense of Humor and Physical Health: Theoretical Issues, Recent Findings, and Future Directions,” Humor 17 (2004): 1–19, 10.1515/humr.2004.005.

[10] K. Buxman , “Humor in the OR: A Stitch in Time?,” AORN Journal 88 (2008): 67–77, 10.1016/j.aorn.2008.01.004. 18603034

[11] S. Sarıtaş , H. Genç , Ş. Okutan , R. İnci , A. Özdemir , and G. Kizilkaya , “The Effect of Comedy Films on Postoperative Pain and Anxiety in Surgical Oncology Patients,” Complementary Medicine Research 26 (2019): 231–239, 10.1159/000497234. 30921794

[12] F. Kurudirek , D. Arikan , and A. Sarialioğlu , “Effects of Therapeutic Clowning on Pain and Anxiety During Venous Blood Sampling in Turkey: Randomised Controlled Trial,” Journal for Specialists in Pediatric Nursing 26 (2021): e12352, 10.1111/jspn.12352. 34216423

[13] J. Cohen , Statistical Power Analysis for the Behavioral Sciences, 2nd ed. (Erlbaum, 1988).

[14] F. Faul , E. Erdfelder , A. Lang , and A. Buchner , “G*Power 3: A Flexible Statistical Power Analysis Program for the Social, Behavioral, and Biomedical Sciences,” Behavior Research Methods 39 (2002): 175–191, 10.3758/bf03193146. 17695343

[15] N. Crıchton , “Visual Analogue Scale (VAS),” Journal of Clinical Nursing 10 (2001): 706.

[16] E. M. Morais , P. R. Moreira , and E. R. Winkelmann , “Movie Watching During Dialysis Sessions Reduces Depression and Anxiety and Improves Quality of Life: A Randomized Clinical Trial,” Complementary Therapies in Medicine 52 (2020): 102488, 10.1016/j.ctim.2020.102488. 32951737

[17] Y. Krieger , M. Pachevsky , Y. Shoham , R. Biederko , L. Novack , and O. Sarid , “Relieving Pain and Distress Symptoms in the Outpatient Burn Clinic: The Contribution of a Medical Clown,” Burns 48 (2022): 654–661, 10.1016/j.burns.2021.06.008. 34670712

[18] N. Meiri , A. Ankri , M. Hamad‐Saied , M. Konopnicki , and G. Pillar , “The Effect of Medical Clowning on Reducing Pain, Crying, and Anxiety in Children Aged 2–10 Years Old Undergoing Venous Blood Drawing—A Randomized Controlled Study,” European Journal of Pediatrics 175 (2016): 373–379, 10.1007/s00431-015-2652-z. 26475347

[19] L. Wang , J. Zhu , and T. Chen , “Clown Care in the Clinical Nursing of Children: A Meta‐Analysis and Systematic Review,” Frontiers in Pediatrics 12 (2024): 1324283, 10.3389/fped.2024.1324283. 38590768 PMC10999578

[20] O. B. Yun , S. J. Kim , and D. Jung , “Effects of a Clown–Nurse Educational Intervention on the Reduction of Postoperative Anxiety and Pain Among Preschool Children and Their Accompanying Parents in South Korea,” Journal of Pediatric Nursing 30 (2015): e89–e99. 25882469 10.1016/j.pedn.2015.03.003

[21] H. Elmali and R. B. Akpinar , “The Effect of Watching Funny and Unfunny Videos on Post‐Surgical Pain Levels,” Complementary Therapies in Clinical Practice 26 (2017): 36–41, 10.1016/j.ctcp.2016.11.003. 28107847

[22] Ü. Fındık and S. Topçu , “Effect of the Way of Surgery on Preoperative Anxiety [Cerrahi Girişime Alınış Şeklinin Ameliyat Öncesi Anksiyete Düzeyine Etkisi],” Shanghai Bu Yi Xue Bao=Shanghai Journal of Immunology 19 (2012): 22–33, https://dergipark.org.tr/en/pub/hunhemsire/issue/7853/103361.

[23] B. Yurddaş and E. S. Ak , “The Effect of Anxiety Levels of Patients Before Laparoscopic Cholecystectomy Surgery on Postoperative Pain and Sleep: Descriptive Research [Laparoskopik Kolesistektomi Ameliyatı Öncesi Hastaların Anksiyete Düzeylerinin Ameliyat Sonrası Ağrı ve Uyku Üzerine Etkisi: Tanımlayıcı Araştırma],” Turkiye Klinikleri Journal of Nursing Sciences 14 (2022): 1000–1008, 10.5336/nurses.2022-91112.

[24] B. C. Gill , L. E. Miller , S. Bhattacharyya , H. Cash , and G. R. Eure , “Complications of Green Light Laser Versus Transurethral Resection of the Prostate for Treatment of Lower Urinary Tract Symptoms: Meta‐Analysis of Randomized Trials,” Urology 184 (2024): 259–265, 10.1016/j.urology.2023.12.018. 38176618

[25] H. Genç and S. Saritas , “The Effects of Watching Comedy Videos on Anxiety and Vital Signs in Surgical Oncology Patients,” Explorer 16 (2020): 401–406, 10.1016/j.explore.2020.02.009. 32247709

